# The Electrical Conductivity of Molten Oxide-Fluoride Cryolite Mixtures

**DOI:** 10.3390/ma14237419

**Published:** 2021-12-03

**Authors:** Pavel Arkhipov, Olga Tkacheva

**Affiliations:** Institute of High Temperature Electrochemistry, Ural Branch of the Russian Academy of Sciences, 620990 Ekaterinburg, Russia; o.tkacheva@ihte.uran.ru

**Keywords:** electrical conductivity, cryolite, alumina, calcium fluoride, activation energy

## Abstract

A new way to reduce the energy consumption during the operation of powerful aluminum reduction cells is suggested via reducing the resistance of the electrolyte, i.e., increasing its electrical conductivity. The electrical conductivity of molten cryolite mixtures NaF-AlF_3_-CaF_2_-Al_2_O_3_ with cryolite ratio (CR) of 2.1–3.0 and content of CaF_2_ and Al_2_O_3_, up to 8 wt%, was measured at the temperatures from liquidus to 1300 K. Based on the experimental results, a multifunctional equation for the electrical conductivity of oxide-fluoride cryolite melts was evaluated. The experimental and calculated values of the electrical conductivity agree within 1.5%. The activation energy of the electrical conductivity of the NaF-AlF_3_-CaF_2_-Al_2_O_3_ melts was estimated. The activation energy of electrical conductivity for molten NaF-AlF_3_ mixtures with CR 3.0 and 2.1, determined by the most mobile cations Na^+^, increased from 15.8 kJ/mol up to 18.5 kJ/mol. It was found that CR had a greater impact on the activation energy than the changes in the Al_2_O_3_ or CaF_2_ concentrations. Based on the ratio of the activation energies of the electrical conductivity and the viscous flow, the correlation between the electrical conductivity and viscosity of molten cryolite mixtures NaF-AlF_3_-CaF_2_-Al_2_O_3_ was illustrated.

## 1. Introduction

A distinctive feature of the primary aluminum electrolytic production is a high specific power consumption. The average specific power consumption for electrolytic cells with baked anodes is 14,200 kWh/t Al, and for cells with self-baking anodes—from 15,300 kWh/t Al (for cells with side current supply) to 16,000 kWh/t (for cells with the upper current lead), and only 40% of this electricity is consumed directly for the aluminum production; the rest is spent on heating the cell and heat losses [1,2]. In connection with the commissioning of powerful 400–500 kA cells and the constantly ongoing modification of the design of existing electrolyzers, there is a great need to solve the issues related to electrical conductivity, thermal conductivity, hydrodynamic, and diffusion processes [3,4,5]. The tendency to raise the cells capacity can be clearly seen on the example of the Chinese aluminum industry. Twenty-seven out of thirty-four large aluminum companies in China use Cell technology 400–500 kA. Two companies, China Hongqiao Group and Shanxi Non-Ferrous Co. Ltd., implement Cell technology 600 kA [6]. The Shandong Xinfa Aluminum and Electricity Group have built three new 660 kA power lines, which are now operating in Liaocheng (Chipin dong province, China). The capacity of the 660 kA aluminum smelter is 1.15 million tons per year [7].

The practical implementation of the obtained results consists of determining the optimal operating modes and design features of electrolysis cells in order to increase their energy efficiency and environmental friendliness. Currently, the work is underway to optimize the thermal characteristics of aluminum cells. Newly proposed principles and models for controlling the thermal characteristics of the cells will improve energy efficiency by more than 50% [1,8]. The main ways of reducing the power consumption during the operation of powerful cells include a reduction in the anode-cathode pole-to-pole distance, decrease in the ohmic resistance in the current-carrying parts of the electrolyzer, and decrease in the electrolyte resistance or, in other words, increase in the electrical conductivity. The alumina addition to the electrolytic cell is an important process in the aluminum electrolysis. The existing mathematical models considering the electrolytic cell feeding process are based on the reference data on physical-chemical properties of cryolite-alumina melts and alumina solubility. These models describe the operation of electrolyzers with medium current load (about 160 kA). However, in powerful electrolyzers exceeding 300 kA, the distribution of alumina concentration in the electrolyte, which is determined by the feeding strategy, influences such technological characteristics as current efficiency, frequency of anode effects, and power consumption. The mathematical model for correlation of physical fields (electrical, electromagnetic, and temperature) of the industrial electrolyzer requires experimental data on electrical conductivity and viscosity of the electrolytes depending on the alumina concentration, temperature, and electrolyte composition.

Conventional electrolytes for aluminum production are based on sodium cryolite. The electrical conductivity of individual sodium cryolite (Na_3_AlF_6_ ≡ 3NaF-AlF_3_) and its mixtures has been well studied [9]. It is established that the electrical conductivity of sodium cryolite near the melting point is 2.83 S·m^−1^·10^−2^. The NaF-AlF_3_ electrolyte is characterized by the parameter of cryolite ratio (CR), which is expressed by the molar ratio of sodium fluoride to aluminum fluoride CR= *n*_NaF_/*n*_AlF3_. An industrial electrolysis, as a rule, is performed at the temperatures of 1223 ± 5 K. A decrease in the liquidus temperature of sodium cryolite is achieved by increasing the AlF_3_ concentration, as well as by addition of CaF_2_, MgF_2_, and Al_2_O_3_. Therefore, typical CR of the electrolyte is 2.3 ± 0.1. A decrease in the operating temperature allows suppressing the secondary reaction of aluminum dissolution and, hence, increases in the current efficiency. An increase in the AlF_3_ concentration in the NaF-AlF_3_ mixture, i.e., a decrease in CR, causes a decrease both in the electrical conductivity and in the alumina solubility in molten cryolites. A review [10] compares the electrical conductivity of the NaF–AlF_3_–Al_2_O_3_ melt at different CR, ranging from 2.2 to 3.0. It can be noted that the lower CR leads to the greater discrepancy in the results obtained by different researchers.

The CaF_2_ additives reduce the electrical conductivity of cryolite melts [11]. The addition of CaF_2_ increases the viscosity of the electrolyte and decreases its electrical conductivity. A decrease in the electrical conductivity is associated with the appearance of complex ions and a decrease in the number of charge carriers. As CR values decreases, the effect of CaF_2_ addition on the electrical conductivity of the cryolite melts becomes more significant. It was found [12] that the CaF_2_ reduced the electrical conductivity of the NaF-AlF_3_ melt more when the proportion of AlF_3_ increased from 10 to 60 wt%, which is explained by the formation of CaAlF_5_ and NaCaAlF_6_ compounds.

The research on the electrical conductivity of fluoride cryolite melts is a complex experimental issue. In addition to the general problems, such as the dependence of the electrolyte resistance on the frequency of alternating current, etc., it is also necessary to take into account the high corrosion activity of fluoride melts and the temperatures of the experiment exceeding 1273 K. To perform a more accurate measurement, coulometric cells with a capillary made of insulating material, which provides sufficiently high electrical resistance, are required. For aggressive fluoride melts, the capillary material, as a rule, is boron nitride (BN) [13,14,15]. However, the BN has poor wettability, which is why it is necessary to increase the diameter of the capillary and select its size individually for each salt. For example, in works [9,16,17], a tube made of pyrolytic BN was used instead of the capillary.

The most resistant material for a cell designed to measure the electrical conductivity of fluoride melts is platinum. Cells with parallel platinum electrodes have been successfully used by Slovak scientists to measure the electrical conductivity of molten sodium and lithium cryolites, and their mixtures, at the temperatures of 1173–1323 K [18]. Cells with parallel electrodes, despite the small value of the constant, in some cases, have real advantages over the capillary cells [14]. The use of cells with parallel electrodes makes it possible (i) to record changes in the electrical conductivity of molten mixtures with the gradual addition of salts or oxides in one experiment; (ii) to measure the electrical conductivity of melts in a wide temperature range, including the temperatures below the liquidus temperature, in the two-phase region; (iii) to work with molten fluoride salts that destroy the capillary material. Usually, tungsten and molybdenum are used as electrodes.

There are several empirical equations that describe the electrical conductivity of molten multicomponent systems based on sodium cryolite, depending on the temperature and concentration of the components [18,19,20,21,22,23]. The field of application of the equations is limited by the concentration and temperature ranges. The values of the electrical conductivity of the Na_3_AlF_6_-AlF_3_ electrolytes with an excess of the AlF_3_ content (more than 15 wt%), calculated using the equations proposed by different authors, vary significantly in the region of high AlF_3_ concentrations. It is necessary to take into account the fact that, when deriving equations for the electrical conductivity of multicomponent systems, as a rule, the experimental data obtained for binary electrolytes are used. With an increase in the number of melt components and their concentrations, the discrepancy between the calculated values increases.

Thus, despite the possibility of predicting the values of the electrical conductivity for molten fluoride mixtures of various compositions, it is still crucial to obtain experimental results, which gives grounds for developing more and more accurate model calculations.

This work is aimed at:

(i) measuring the electrical conductivity of fluoride-oxide cryolite melts NaF-AlF_3_-CaF_2_-Al_2_O_3_ in a wide range of compositions (CR 2.1–3.0, CaF_2_ and Al_2_O_3_ concentrations ranging from 0 to 8 wt%) in the temperature range from liquidus to 1300 K;
(ii) obtaining a multifunctional equation for calculating the electrical conductivity of oxide-fluoride cryolite melts, based on the experimental results;
(iii) estimating the activation energy of electrical conductivity at 1200–1336 K and considering the relationship between the electrical conductivity and the viscosity of oxide-fluoride melts.

## 2. Materials and Methods

The main component, sodium cryolite NaF-AlF_3_, was prepared from individual salts of AlF_3_ and NaF (Vekton, CJSC, St. Petersburg, Russia). The required amount of components (depending on CR) was placed in a graphite container and was heated according to the following heating mode:

(1) up to 493 K and exposed for 2 h to remove moisture;
(2) up to 773 K and exposed for 2 h to remove water of crystallization;
(3) up to 1323 K until the electrolyte is completely melted.
(4) cooling to room temperature.

The chemically pure CaF_2_ and Al_2_O_3_ components (Vekton, CJSC, St. Petersburg, Russia) were added to the prepared mixture NaF-AlF_3_ during measurement.

The prepared mixture was analyzed for the content of Al, Na, and O. The latter was determined using an oxygen analyzer LECO OH 836 (LECO, St. Joseph, MI, USA), which is based on carbothermal oxygen reduction in solid samples. The Al and Na concentrations are determined by an ICP-MC device (CiCAP 6300 Duo, Thermo Scientific, Waltham, MA, USA). If necessary, the composition of the NaF-AlF_3_ mixture was adjusted to the required CR value by adding AlF_3_.

**Figure 1:**
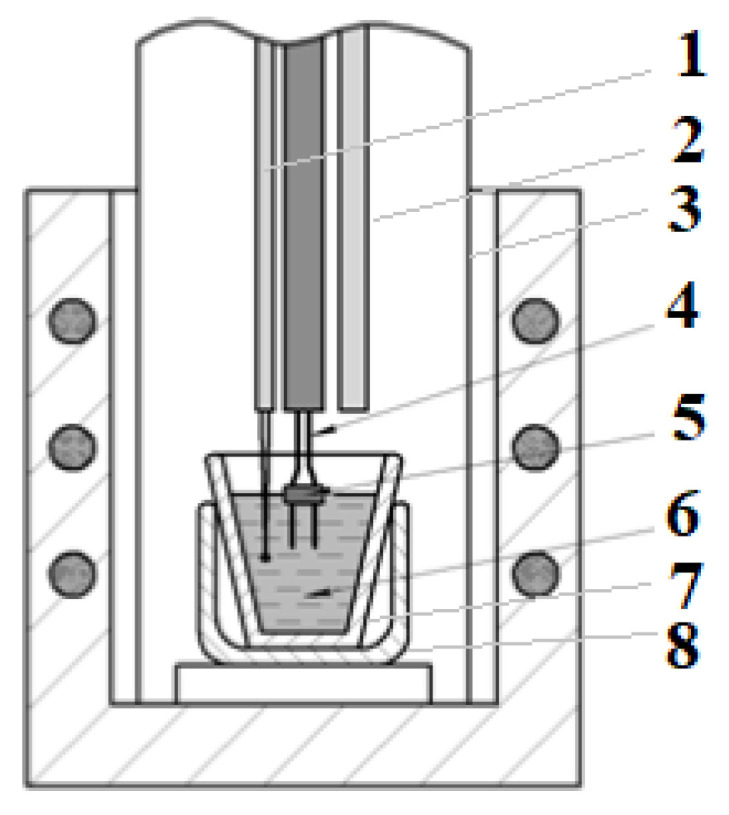
Schematic of cell with parallel electrodes for measuring the electrical conductivity: 1—Pt/Pt (Rh) thermocouple, 2—additive tube (alumina), 3—quartz container, 4—molybdenum electrodes, 5—BN-bar, 6—melt, 7—glass carbon crucible, 8—supporting guard crucible (alumina).

The electrical conductivity was measured by the electrochemical impedance spectroscopy (EIS) using a PGSTAT AutoLab 302N (Eco Chemie, Metrohm Autolab B.V., Utrecht, The Netherlands) in an electrochemical cell, with two parallel electrodes immersed into the melt. The cell layout is shown in Figure 1.

An argon atmosphere was maintained in the cell. The electrolyte resistance was measured by a PGSTAT AutoLab 302N device (Eco Chemie, Metrohm Autolab B.V., Utrecht, The Netherlands). The procedure is described in detail elsewhere [14]. A distinctive feature of the cell is the use of a boron nitride bar. This bar provided a rigid fixation of the electrodes and a constancy of the surface area of the electrodes in the melt. Thus, a change in the volume of the melt, when adding CaF_2_ and Al_2_O_3_ during measurements, affected neither the immersion depth of the electrodes, nor the inter electrode distance, which could change due to thermal expansion at the temperatures of about 1323 K.

**Figure 2:**
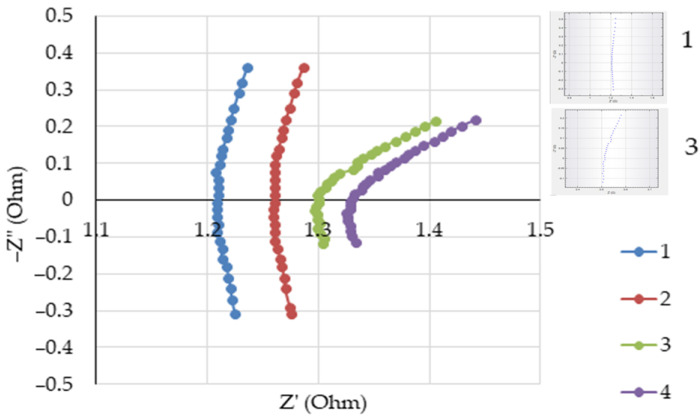
Nyquist plots obtained at 1273 K in the melts: 1—NaF-AlF_3_ (CR = 2.3); 2—NaF-AlF_3_ (CR = 2.1); 3—NaF-AlF_3_ (CR = 2.1) + 2 wt% Al_2_O_3_; 4—NaF-AlF_3_ (CR = 2.1) + 4 wt% Al_2_O_3_. The Autolab original plots for melts 1 and 3 are embedded.

The melt resistance was determined using the Nyquist plot, according to the value of the active part of the impedance in the point of the X-axis intersection. As an example, Nyquist plots for some melts are shown in Figure 2. Curves 1 and 2 were obtained in the frequency range from 100 to 4 kHz, and curves 3 and 4 were obtained at the frequencies ranging from 50 to 4 kHz.

The electrical conductivity was calculated according to the formula:

*κ* = K/R (1)

where *κ* is the specific electrical conductivity (S/m·10^−2^), K is the cell constant (1/m·10^−2^), and R is the ohmic resistance of the electrolyte (Ohm).

The cell constant was determined from the known value of the electrical conductivity of the sodium cryolite melt with CR = 3.0 [9] in the temperature range of 1290–1340 K. The dependence of the constant (K) of the experimental cell, with parallel electrodes on temperature, is described by a linear equation:

K = 1.633 + T·10^−3^ (2)

where T is the temperature, K.

When calculating the electrical conductivity, the dependence of the cell constant on temperature was taken into account according to Equation (2). The calculation error (systematic and random) in measuring the electrical conductivity was 5%.

## 3. Results and Discussion

### 3.1. Measurement of Electrical Conductivity

The electrical conductivity was measured in molten mixtures based on sodium cryolite with different CR. The CR value was changed from 2.1 to 3.0. The concentrations of CaF_2_ and Al_2_O_3_ additions varied from 0 to 8 wt%. The measurement temperature changed from 1336 K (max.) to the temperature of the mixture’s liquidus. The liquidus temperature was calculated using the equation given by Solhiem [22]. The electrolyte composition and the measured conductivity at different temperatures are summarized in Supplementary Materials.

The electrical conductivity of all cryolite mixtures decreases with cooling and decreasing CR (i.e., a decreasing NaF concentration) and with the increasing content of CaF_2_ and Al_2_O_3_.

**Figure 3:**
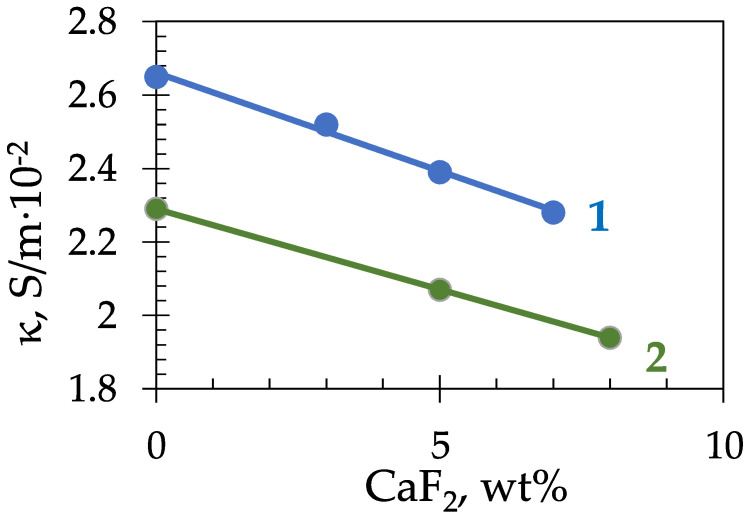
Electrical conductivity of the NaF-AlF_3_-CaF_2_ melts with CR = 2.7 (1) and 2.1 (2) at 1273 K.

The dependence of the electrical conductivity of cryolite melts on the CaF_2_ content is shown, in Figure 3, for the NaF-AlF_3_-CaF_2_ compositions with CR = 2.1 and 2.7.

Based on the results obtained, it can be concluded that, on average, the addition of 1 wt% of CaF_2_ reduces the electrical conductivity of the NaF-AlF_3_ melt by 0.05 S/(m·10^−2^).

**Figure 4:**
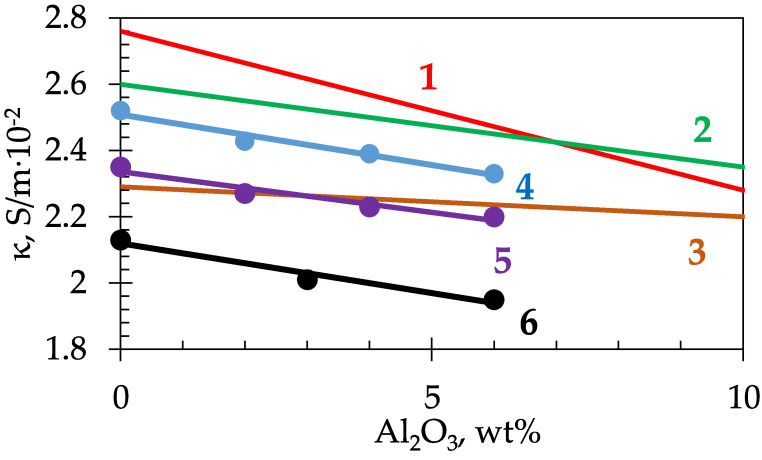
Electrical conductivity of cryolite melts, depending on the alumina concentration at T = 1273 K: 1—NaF-AlF_3_, CR = 3 [24]; 2—NaF-AlF_3_, CR = 2.4 [18]; 3—NaF-AlF_3_, CR = 1.8 [18]; 4—NaF-AlF_3_, CR = 2.4; 5—NaF-AlF_3_, CR = 2.1; 6—NaF-AlF_3_-(5 wt%) CaF_2_, CR = 2.1.

The effect of alumina concentration on the electrical conductivity of cryolite melts can be determined from Figure 4. This figure includes the results, obtained by calculating and using the regression equations, given in [18,24] for the NaF-AlF_3_-Al_2_O_3_ melts with CR 3.0, 2.4, and 1.8 and the newly obtained experimental data for the molten systems: NaF-AlF_3_ with CR = 2.4 and 2.1; NaF-AlF_3_-(5 wt%) CaF_2_ with CR = 2.1.

First, it should be noted that the experimental and calculated data, according to the generalized equation [18] for the electrical conductivity, of the NaF-AlF_3_ (CR 2.4) melt diverge within 4%.

The alumina additions to the cryolite melts, with any CR, reduce its electrical conductivity, which is associated with the appearance of low-mobile oxide-fluoride complex anions of different compositions. According to papers [18,24], the lower values of CR result in the smaller influence of alumina concentration on the electrical conductivity. For example, for cryolite melts with CR = 3.0, the addition of 1% of Al_2_O_3_ decreases the electrical conductivity by 0.05 S/(m·10^−2^) (Figure 4). In NaF-AlF_3_ melts, with CR 2.4 and 1.8, the decrease is 0.025 and 0.01 S/(m·10^−2^), respectively. The different effect of alumina on the change in the electrical conductivity of the NaF-AlF_3_ melts is a consequence of various reactions occurring in neutral and acidic electrolytes during the Al_2_O_3_ dissolution. In neutral cryolite melts (CR = 3) the reaction proceeds as follows:

2AlF_6_^3−^ + 2 Al_2_O_3_ = 3 Al_2_O_2_F_4_^2−^ (3)

In the cryolite melts with low CR the following reactions can occur:

4AlF_6_^3−^ + Al_2_O_3_ = 3 Al_2_OF_6_^3−^ + 6F^−^ (4)

4AlF_4_^−^ + 2Al_2_O_3_ = 3 Al_2_O_2_F_4_^3−^ + 4F^−^ (5)

It follows from reactions (4) and (5) that the alumina dissolution in the NaF-AlF_3_ cryolites with low CR increases the concentration of fluoride ions in the solution, which contributes to the electrical conductivity increase.

For the NaF-AlF_3_ melts, with CR in the range of 2.1–2.7, studied in this work, the change in electrical conductivity with the addition of 1% of Al_2_O_3_ is averagely (0.04–0.05) S/(m·10^−2^). Thus, no significant difference was observed in the slopes of the straight lines describing the dependence of the electrical conductivity (for cryolites with CR ranging from 2.1 to 2.7) on the alumina content. The addition of 5% of CaF_2_ to the NaF-AlF_3_-Al_2_O_3_ melt with CR = 2.1 also had no effect on the curves slope. The results obtained correlate with the data reported by Kubinakova et al. [25], where the decrease in the electrical conductivity of the NaF-AlF_3_ system within the CR range of 1.2–2.0, with the increasing alumina concentration, was about (0.04–0.06) S/(m·10^−2^) per 1 wt% of Al_2_O_3_.

There is another approach to explain the change in the electrical conductivity caused by the alumina addition. Rolin [26] elucidates that the electrical conductivity of sodium cryolite (CR = 3.0 and 2.7) decreases as the alumina content increases, due to the higher viscosity of the melt, which is explained by the appearance of complex anions [Al_2_OF_6_]^2−^ and [Al_2_OF_8_]^4^ anions in the melt. The number of these complex anions grows as the alumina concentration in the melt increases.

### 3.2. Multifunctional Conductivity Equation

Experimental data on the electrical conductivity of molten salt mixtures, as a rule, are presented in the form of empirical equations, reflecting its dependence on the concentration and temperature. These dependences can be expressed by both linear and logarithmic equations:

κ = A + BT(6)

κ =A_κ_·exp(−E_el_/RT) (7)

where κ is the specific electrical conductivity, S/(m·10^−2^), Aκ is the coefficient related to chemical composition of melt; E_el_ is the activation energy of the electrical conductivity, J/mol; T is the temperature, K; R is the universal gas constant, 8.314 J/(mol·K).

These equations are valid in certain concentration and temperature ranges. Nevertheless, it is possible to generalize the electrical conductivity temperature dependence of cryolite mixtures, with different content of components, by one multifunctional equation in wide temperature and concentration ranges. Such calculations were carried out using the Origin program, specially developed for the analysis of experimental data.

The general regression equation for the electrical conductivity of cryolite systems on several parameters was derived by the multivariable data approximation. The data set included the following parameters: temperature, cryolite ratio, calcium fluoride, and alumina content (see Supplementary Materials). The resulting equation has the following form:

κ = −1.87 + 3.23·10^−3^·T − 2.99·10^−2^·C (Al_2_O_3_) + 4.70·10^−1^·CR − 4.37·10^−2^·C (CaF_2_) (8)

where κ is the electrical conductivity, S/(m·10^−2^), T is the temperature, (1200–1336 K), CR is the cryolite ratio, C(CaF_2_) and C(Al_2_O_3_) are the concentrations of additives, wt%. Approximation reliability R^2^ = 0.97 at the temperatures ranging from 1200 K to 1336 K.

**Figure 5:**
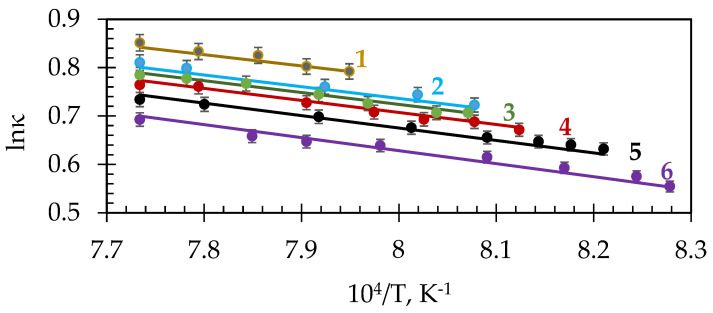
Electrical conductivity of cryolite melts: points denote experimental results, lines denote calculation results (Equation (8)) (wt%): 1—NaF-AlF_3_ CR = 2.5, C (Al_2_O_3_) = 2, C (CaF_2_) = 5; 2—CR = 2.3, C (Al_2_O_3_) = 2, C (CaF_2_) = 5; 3—CR = 2.5, C (Al_2_O_3_) = 6, C (CaF_2_) = 5; 4—CR = 2.3, C (Al_2_O_3_) = 4, C (CaF_2_) = 5; 5—CR = 2.1, C (Al_2_O_3_) = 3, C (CaF_2_) = 5; 6—CR = 2.1, C (Al_2_O_3_) = 6, C (CaF_2_) = 5.

As an example, the experimental and calculated, by Equation (8) values of the electrical conductivity of oxide-fluoride NaF-AlF_3_-CaF_2_-Al_2_O_3_ melts, are plotted in Figure 5.

The calculated electrical conductivity is presented as solid lines, and the experimentally obtained values of electrical conductivity are illustrated in the form of points. The experimental and calculated values of the electrical conductivity agree within 1.5%.

### 3.3. Activation Energy of Electrical Conductivity

The electrical conductivity of cryolite melts in coordinates lnκ—*f* (T) is described by the following equation:

lnκ = A + B/T(9)

The values of A and B coefficients of Equation (9), for some compositions of cryolite mixtures, and the calculated activation energy of the electrical conductivity are presented in Table 1.

The molten cryolite NaF-AlF_3_ systems are ionic melts; their main structural elements are cations Na^+^ and complex anions AlF_6_^3−^, AlF_5_^2−^, AlF_4_^−^ [27,28]. The electrical conductivity of these melts is determined by the most mobile Na^+^ cations. With a decrease in the concentration of sodium cations in the melt, i.e., with CR decrease, the electrical conductivity drops abruptly and, accordingly, the activation energy of this process rises. The additions of Al_2_O_3_ and CaF_2_ also increase the activation energy of the electrical conductivity, due to the formation of complex anions during their dissolution, which impede the charge transfer process. Evaluating E_el_ (Table 1), one can conclude that, for the considered range of Al_2_O_3_ or CaF_2_ concentrations, CR has a greater impact on E_el_ than the Al_2_O_3_ or CaF_2_ concentrations changes. The E_el,_ values of molten NaF-AlF_3_ increases from 15.8 kJ/mol at CR = 3 to 18.5 kJ/mol at CR = 2.1. The aforesaid illustrates the structural changes in the molten NaF-AlF_3_ fluoride system, i.e., the number of mobile Na^+^ ions decreases and the number of AlF_6_^3−^, AlF_5_^2−^, AlF_4_^−^ complex anions increases. The increasing Al_2_O_3_ and CaF_2_ concentrations decrease the electrical conductivity under otherwise equal conditions. The values of the activation energy of electrical conductivity of molten NaF-AlF_3_ increase to 20.0 kJ/mol at 6 wt.% of Al_2_O_3_ and up to 22.5 kJ/mol at 8 wt.% of CaF_2_. Comparing the E_el_ values presented in Table 1, we may conclude that, within the analyzed range of changes in the molten salt compositions, the value of CR, i.e., decrease in the NaF in the melt, has the greatest impact has the greatest impact on the E_el_ value.

On the other hand, transport properties, such as the melt viscosity, also change exponentially with temperature:

η = Aη·exp (E_vis_/RT) (10)

However, the viscosity is determined by the mobility of complex anions. Even though the mechanism of the electrical conductivity and the viscous fluid flow are different, there is a certain relationship between them. The relationship between the electrical conductivity and the viscosity for molten salts was proposed by Frenkel [29]:

κ^n^·η = Const (11)

where *n* = E_vis_/E_el_.

Here *η* is the dynamic viscosity ((Pa·10^−3^)·s), *κ* is the electrical conductivity (S/(m·10^−2^)), and E_vis_ is the activation energy of the viscous flow (kJ/mol).

The activation energy of viscosity for some oxide-fluoride cryolite melts is given in Table 2. It was calculated using the equation for the viscosity proposed in our papers [30,31]:

η = 71.75 − 1.33·10^−1^·(T − 273) − 8.21·10^−3^·C (Al_2_O_3_) + 3.33·10^−1^·CR + 0.0796·C (CaF_2_) −0.625·10^−5^·T^2^ − 2.08·((C (CaF_2_)+ C (Al_2_O_3_))/C (Al_2_O_3_)^1.5^ + 8.12·10^−5^·C (Al_2_O_3_)^3^ (12)

where T is the temperature (K), C (Al_2_O_3_), and C (CaF_2_) are the concentrations of components (wt%), the CR is the cryolite ratio. The equation is true in the temperature range from the liquidus to 1300 K, CR ranges from 2.1 to 2.5, the Al_2_O_3_ and CaF_2_ content is up to 6 and 8 wt%, respectively.

A decrease in the CR of the NaF-AlF_3_ system, and the addition of the Al_2_O_3_ and CaF_2_ to the melt, lead to an increase in the activation energy of the viscous flow.

The ratios between the electrical conductivity and the dynamic viscosity, calculated according to Equation (11), for some cryolite compositions are presented in Table 2.

Table 2 elucidates that, for the illustrated composition of the molten mixtures, the experimentally obtained values of electrical conductivity increase as the temperature increases, and the values of viscosity, calculated for the identical conditions, decrease as the temperature increases. The activation energy of the electrical conductivity increases as CR increases, and Al_2_O_3_ and CaF_2_ are added to the electrolyte. The activation energy of viscous flow increases as the CR, Al_2_O_3_, and CaF_2_ concentrations increase. The ratio (*n*) of the activation energy of viscous flow to the electrical conductivity activation energy is greater than unity because the electrical transfer is performed by the ions of smaller size, and the viscous flow is determined by the particles of the larger size. The product of (κ^n^·η) is a constant for a given melt composition and does not depend on temperature. This fact testifies the ionic nature of the melt.

## 4. Conclusions

The experimental study of the electrical conductivity of the fluoride-oxide cryolite mixtures NaF-AlF_3_-CaF_2_-Al_2_O_3_ was carried out in a wide range of CaF_2_ and Al_2_O_3_ compositions (0–8 wt%) and CR ranging from 2.1 to 3.0 in the temperature range from liquidus to 1300 K. A multifunctional equation for calculating the electrical conductivity of oxide-fluoride melts based on sodium cryolite was evaluated.

The electrical conductivity of the molten NaF-AlF_3_ system was found to increase as the temperature and the cryolite ratio increased, whereas the content of alumina and calcium fluoride decreased.

The activation energy of electrical conductivity for molten NaF-AlF_3_ mixtures increases from 15.8 kJ/mol (at CR = 3) up to 18.5 kJ/mol (at CR = 2.1). This indicates the structural changes in the molten fluoride system towards a decrease in the number of mobile cations Na^+^, and an increase in the content of complex anions AlF_6_^3−^, AlF_5_^2−^, AlF_4_^−^.

An increase in the content of CaF_2_ and Al_2_O_3_ decreases the electrical conductivity, under otherwise equal conditions. The activation energy of electrical conductivity of molten NaF-AlF_3_-CaF_2_-Al_2_O_3_ mixtures increases to 20.0 kJ/mol (at 6 wt% of Al_2_O_3_) and up to 22.5 kJ/mol (at 8 wt% of CaF_2_). Electrical conductivity depends more on the CaF_2_ content than on alumina at the same temperature and cryolite ratio.

Based on the ratio of the activation energies of the electrical conductivity and the viscous flow, the correlation between the electrical conductivity and viscosity of molten cryolite mixtures NaF-AlF_3_-CaF_2_-Al_2_O_3_ was illustrated.

## Figures and Tables

**Table 1:** Coefficients A and B for lnκ = A + B/T and the activation energy of the electrical conductivity of cryolite melts.

	A	−B, K^−1^	R^2^	E_el,_ kJ/mol	T_liq_—T, K
		NaF-AlF_3_			
**CR**					
3.0	2.5123	1901	99.8	15.8	1281–1336
2.5	2.5546	2068	99.9	17.2	1277–1293
2.3	2.5747	2142	99.8	17.8	1258–1293
2.1	2.6001	2224	99.8	18.5	1244–1293
		NaF-AlF_3_ CR = 2.1			
**Al_2_O_3_**					
2	2.6224	2285	99.9	19.0	1222–1293
4	2.6448	2347	99.8	19.5	1210–1293
6	2.6693	2413	99.8	20.0	1200–1293
		NaF-AlF_3_ CR = 2.1			
**CaF_2_**					
5	2.7148	2531	99.8	21.0	1228–1293
8	2.7863	2705	99.8	22.5	1225–1293

**Table 2:** Relationship between the electrical conductivity and the viscosity of cryolite melts.

	T, K	κ, S/(m·10^−2^)	η, (Pa·10^3^)·s	κ^n^·η
	NaF-AlF_3_, CR = 2.3, E_el_ = 17.80 kJ/mol, E_vis_ = 41.94 kJ/mol, *n* = 2.36			
1	1293	2.51	2.38	20.8
2	1383	2.48	2.44	20.8
3	1273	2.45	2.51	20.7
4	1263	2.41	2.59	20.6
	NaF-AlF_3_, CR = 2.1, E_el_ = 18.48 kJ/mol, E_vis_ = 45.08 kJ/mol, *n* = 2.44			
5	1293	2.41	2.32	19.9
6	1283	2.38	2.38	19.7
7	1273	2.35	2.45	19.6
8	1263	2.31	2.53	19.6
9	1253	2.28	2.62	19.7
10	1243	2.25	2.73	19.7
	NaF-AlF_3_, CR = 2.1, Al_2_O_3_ = 2 wt%, E_el_ = 19.00 kJ/mol, E_vis_ = 45.89 kJ/mol, *n* = 2.42			
11	1293	2.35	2.26	18.1
12	1383	2.32	2.31	17.9
13	1273	2.29	2.38	17.8
14	1263	2.25	2.46	17.8
15	1253	2.22	2.55	17.8
16	1243	2.19	2.66	17.9
17	1233	2.16	2.78	18.0
	NaF-AlF_3_, CR = 2.1, Al_2_O_3_ = 4 wt%, E_el_ = 19.50 kJ/mol, E_vis_ = 46.33 kJ/mol, *n* = 2.38			
18	1293	2.29	2.26	16.2
19	1283	2.26	2.31	16.1
20	1273	2.23	2.38	16.0
21	1263	2.20	2.46	16.0
22	1253	2.16	2.55	16.0
23	1243	2.13	2.66	16.1
24	1233	2.10	2.78	16.2
	NaF-AlF_3_, CR = 2.1, CaF_2_ = 5 wt%, Al_2_O_3_ = 2 wt%, E_el_ = 21.03 kJ/mol, E_vis_ = 50.21 kJ/mol, *n* = 2.39			
25	1293	2.13	2.52	15.4
26	1283	2.10	2.60	15.3
27	1273	2.07	2.67	15.2
28	1263	2.04	2.75	15.0
29	1253	2.00	2.84	15.0
30	1243	1.97	2.95	14.9
31	1233	1.94	3.07	14.9

Uncertainties (u) are equal to *u (T)* = 0.5 K, u (κ) = 0.01 S/(m·10^−2^), u (η) = 0.01·10^−3^ Pa·s.

## Data Availability

All data are freely available.

## Supplementary Materials

The following are available online at https://www.mdpi.com/article/10.3390/ma14237419/s1, Table S1: Composition and the electrical conductivity of the NaF-AlF_3_-CaF_2_-Al_2_O_3_ melts.

## References

[1] Gorlanov E.S., Brichkin V.N., Polyakov A.A. (2020). Electrolytic production of aluminium. Review. Part 1. Conventional areas of development. Tsvetnye Metally.

[2] Gorlanov E.S., Kawalla R., Polyakov A.A. (2020). Electrolytic production of aluminium. Review. Part 2. Development Prospects. Tsvetnye Metally.

[3] Dingxiong L., Yungang B., Junman Q., Zijin A., Lindsay S.J. (2011). New progress on application of NEIU400kA family high energy efficiency aluminium reduction pot «HEEP» technology. Light Metals.

[4] Thibeault P., Mezin H., Martin O., Williams E. (2016). Rio Tinto AP44 cell technology development at ALMA smelter. Light Metals.

[5] Meijer M., Johnson J.A. (2010). New logistic concept for 400 and 500 kA smelters. Light Metals.

[6] Gao B., Wang Z., Shi Z., Hu X. History and recent developments in aluminum smelting in China. Travaux 46. Proceedings of the 35th International ICSOBA Conference.

[7] Tabereaux A. (2017). Super-high amperage prebake cell technologies in operation at worldwide aluminum smelters. Light Met. Age.

[8] Liang X., Perander L. (2021). Optimization of thermal characteristics and “Output side energy saving” of aluminum reduction cell. Light Metals.

[9] Hives J., Thonstad J., Sterten A., Fellner P. (1996). Electrical conductivity of molten cryolite-based mixtures obtained with a tube-type cell made of pyrolytic boron nitride. Metall. Mater. Trans. B.

[10] Cassayre L., Palau P., Chamelot P., Massot L. (2010). Properties of low-temperature melting electrolytes for the aluminum electrolysis process: A review. J. Chem. Eng. Data.

[11] Fellner P., Midtlyng S., Sterten A., Thonstad J. (1993). Electrical conductivity of low melting baths for aluminium electrolysis: The system Na_3_AlF_6_–Li_3_AlF_6_–AlF_3_ and the influence of additions of Al_2_O_3_, CaF_2_ and MgF_2_. J. Appl. Electrochem..

[12] Dedyukhin A., Tkacheva O., Redkin A., Zaikov Y., Polyakova M. (2017). Calcium Fluoride Effect on the Physical-Chemical Properties of Cryolites Prospective for Low-Temperature Aluminum Electrolysis, Proceedings of the AIP Conference, St. Petersburg, Russia, 19–21 May 2017.

[13] Moran E. (2017). Boron Nitride: Properties, Synthesis and Applications.

[14] Kryukovsky V.A., Frolov A.V., Tkacheva O.Y., Redkin A.A., Zaikov Y.P., Khokhlov V.A., Apisarov A.P., Galloway T.J. (2006). Electrical Conductivity of Low Melting Cryolite Melts, Proceedings of the 135th TMS Annual Meeting, San Antonio, TX, USA, 12–16 March 2006.

[15] Palimąka P., Pietrzyk S., Sak T. (2016). Application of CVCC technique for measuring electrical conductivity of metallurgical slags and molten salts. Key Eng. Mater..

[16] Huang Y., Lai Z., Tian J., Li J., Liu Y., Li Q. (2008). Electrical conductivity of (Na_3_AlF_6_–40 wt.%K_3_AlF_6_)–AlF_3_ melts. J. Cent. South. Univ..

[17] Yang J., Li W., Yan H., Liu D., Sadler B.A. (2013). Conductivity of KF–NaF–AlF_3_ system low-temperature electrolyte. Light Metals.

[18] Chrenkova M., Danek V., Silny A., Utigard T., Hale W. (1996). Density, Electrical Conductivity and Viscosity of Low Melting Baths for Aluminum Electrolysis, Proceedings of the 125th TMS Annual Meeting, Anaheim, CA, USA, 4–8 February 1996.

[19] Fellner P., Kobbeltvedt O., Sterten A., Thonstad J. (1993). Electrical conductivity of molten cryolite-based binary mixtures obtained with a tube-type tell made of pyrolytic boron nitride. Electrochem. Acta.

[20] Wang X., Peterson R.D., Tabereaux T., Das S.K. (1993). A Multiple Regression Equation for the Electrical Conductivity of Cryolite Melts, Proceedings of the 122nd TMS Annual Meeting, Denver, CO, USA, 21–25 February 1993.

[21] Redkin A.A., Tkacheva O.Y., Shuryghin A.P., DeYoung D.H. (2008). Electrical Conductivity of Molten Electrolytes for Light Metal Production, Proceedings of the 137th TMS Annual Meeting, New Orleans, LO, USA, 9–13 March 2008.

[22] Solheim A., Rolseth S., Skybakmoen E. (1996). Liquidus temperatures for primary crystallization of cryolite in molten salt systems of interest for aluminum electrolysis. Metall. Mater. Trans. B.

[23] Híveš J., Fellner P., Thonstad J. (2013). Transport numbers in the molten system NaF–KF–AlF_3_–Al_2_O_3_. Ionics.

[24] Grjotheim K., Krohn C., Malinovsky M., Matiasovsky K., Thonstad J. (1982). Aluminium Electrolysis. Fundamentals of the Hall-Heroult Process.

[25] Kubinakova E., Danielik V., Hıves J. (2017). Electrical conductivity of low-temperature cryolite electrolytes with high addition of aluminum fluoride. JES.

[26] Rolin M. (1972). Conductivite electrique des melanges a base de cryolithe fondue: Systemes NaF-AlF_3_, AlF_6_Na_3_–Al_2_O_3_ et AlF_6_Na_3_–CaF_2_. Electrochim. Acta.

[27] Parker S.F., Ramirez-Cuesta A.J., Daemen L.L. (2020). The structure and vibrational spectroscopy of cryolite, Na_3_AlF_6_. RSC Adv..

[28] Lacassagne V., Bessada C., Florian P., Bouvet S., Ollivier B., Coutures J.-P., Massiot D. (2002). Structure of high-temperature NaF–AlF_3_–Al_2_O_3_ Melts: A multinuclear NMR study. J. Phys. Chem. B.

[29] Frenkel J. (1955). Kinetic Theory of Liquids.

[30] Lyutina A.S., Kataev A.A., Rudenko A.V., Tkacheva O.Y. (2021). Effect of Al_2_O_3_ and CaF_2_ additives on the viscosity of conventional cryolite melts. Chim. Techno Acta.

[31] Tkacheva O., Arkhipov P., Kataev A., Rudenko A., Zaykov Y. (2021). Electrolyte viscosity and solid phase formation during aluminium electrolysis. Electrochem. Commun..

