# Supplementary material for: The Electrical Conductivity of Molten Oxide-Fluoride Cryolite Mixtures
Source: Materials (Basel). 2021 Dec 3;14(23):7419. doi: 10.3390/ma14237419 (PMC8658855; doi:10.3390/ma14237419)
Supplement: Supplementary file 1 [file materials-14-07419-s001.zip › materials-1444161-supplementary.pdf]

The electrical conductivity of molten oxide-fluoride cryolite mixtures

Pavel Arkhipov and Olga Tkacheva

Table S1. Composition and the electrical conductivity of the $NaF-AlF_3-CaF_2-Al_2O_3$ melts

	Composition			T_{liq}, K	κ, S/m·10^{-2}	T, K
	CR	CaF_2, wt%	Al_2O_3, wt%			
1	3	0	0	1281	2.95	1330
2					2.94	1326
3					2.93	1320
4					2.91	1314
5					2.89	1308
6					2.84	1293
7					2.80	1283
8	2.7	0	0	1267	2.82	1344
9					2.82	1341
10					2.82	1340
11					2.79	1330
12					2.76	1320
13					2.73	1309
14					2.70	1299
15					2.65	1277
16					2.68	1288
17					2.64	1270
18					2.62	1268
19	2.7	5	0	1234	2.65	1354
20					2.63	1351
21					2.62	1345
22					2.58	1342
23					2.56	1338
24					2.50	1316
25					2.47	1306
26					2.45	1297
27					2.43	1288
28					2.39	1278
29					2.36	1270
30	2.7	7	0	1263	2.45	1334
31					2.44	1325
32					2.42	1319
33					2.40	1312
34					2.38	1303
35					2.35	1294
36					2.31	1285
37					2.28	1276
38					2.25	1268
39	2.5	0	0	1277	2.60	1293
40					2.57	1283

41					2.54	1277
42	2.5	5	2	1250	2.34	1293
43					2.30	1283
44					2.28	1273
45					2.23	1265
46					2.21	1258
47	2.5	5	6	1230	2.19	1293
48					2.18	1285
49					2.15	1275
50					2.10	1263
51					2.07	1255
52					2.03	1244
53					2.03	1239
54	2.4	0	0	1255	2.55	1293
55					2.52	1285
56					2.49	1275
57					2.46	1263
58					2.55	1255
59	2.4	7	4	1217	2.36	1293
60					2.33	1288
61					2.26	1269
62					2.18	1252
63					2.17	1255
64					2.16	1249
65					2.11	1236
66					2.09	1226
67					2.05	1217
68	2.4	7	8	1196	2.29	1323
69					2.27	1320
70					2.23	1315
71					2.21	1310
72					2.20	1302
73					2.18	1293
74					2.13	1285
75					2.10	1276
76					2.06	1268
77					2.04	1259
78					2.00	1251
79					1.99	1244
80					1.97	1235
81					1.93	1226
82					1.88	1208
83					1.82	1196
84	2.3	0	0	1258	2.47	1283
85					2.44	1273
86					2.41	1263
87					2.38	1258
88	2.3	5	0	1248	2.22	1285
89					2.14	1262
90					2.10	1248
91	2.3	5	2	1234	2.20	1283
92					2.16	1273
93					2.13	1263

94					2.10	1253
95	2.3	5	4	1223	2.39	1293
96					2.35	1283
97					2.32	1273
98					2.29	1263
99					2.26	1253
100					2.22	1243
101					2.19	1233
102	2.3	5	6	1211	2.15	1293
103					2.14	1283
104					2.07	1265
105					2.03	1254
106					2.00	1246
107					1.99	1238
108					1.96	1235
109	2.1	0	0	1244	2.39	1282
110					2.33	1263
111					2.25	1248
112	2.1	5	0	1236	2.19	1293
113					2.16	1283
114					2.13	1273
115					2.10	1263
116					2.06	1253
117					2.03	1243
118					2.00	1236
119	2.1	5	3	1213	2.08	1293
120					2.06	1282
121					2.01	1263
122					1.97	1248
123					1.93	1236
124					1.91	1228
125					1.90	1223
126					1.88	1218
127	2.1	5	6	1197	2.08	1293
128					2.06	1282
129					2.01	1263
130					1.97	1248
131					1.93	1236
132					1.91	1228
133					1.90	1223
134					1.88	1218
